# Modulation of Presynaptic Inhibitory Retinal Circuits for Retinal Ganglion Cell Neuroprotection in Glaucoma

**DOI:** 10.1002/advs.78179

**Published:** 2026-10-06

**Authors:** Eunji Hong, Chen Lin, Wenjie Long, Ian Kim, Mengwen Xiong, Zuer Wu, Siyuan Rao, Qianbin Wang

**Affiliations:** ^1^ Department of Biomedical Engineering Binghamton University State University of New York Binghamton New York USA; ^2^ Integrative Neuroscience Program Binghamton University State University of New York Binghamton New York USA

**Keywords:** glaucoma, inhibitory retinal circuits, Lin28, ocular hypertension, retinal ganglion cells

## Abstract

Glaucoma is a leading cause of irreversible blindness, yet the circuit‐level mechanisms contributing to retinal ganglion cell (RGC) degeneration remain incompletely defined. Although multiple neuroprotective strategies have been explored, clinical translation remains limited. Here, we identify early dysfunction of inner retinal inhibitory circuitry as a modifiable contributor to RGC vulnerability during ocular hypertensive stress. In mouse ocular hypertension models, inner nuclear layer neurons exhibited increased c‐fos expression and enhanced oscillatory potential‐associated differential electroretinography signals before substantial RGC loss was detected, suggesting early retinal circuit hyperactivity. Broad pharmacological attenuation of GABAergic and glycinergic inhibitory signaling improved RGC survival without reducing intraocular pressure, supporting a functional contribution of inhibitory neurotransmission to RGC vulnerability. Moreover, Cre‐dependent AAV delivery of Lin28, an RNA‐binding regulator of post‐transcriptional gene expression, to Vgat‐positive inhibitory interneurons increased RGC survival compared with Vgat‐EGFP controls and Vglut2‐lineage Lin28 expression. Vgat‐targeted Lin28 also reduced c‐fos expression and oscillatory potential‐associated differential electroretinogram abnormalities, indicating suppression of ocular hypertension‐associated inner retinal hyperactivity. In addition, ocular hypertension was associated with altered sleep architecture, and Vgat‐targeted Lin28 expression was associated with mitigation of these alterations. These findings position presynaptic inhibitory retinal circuitry as an early and modifiable contributor to glaucomatous RGC vulnerability.

## Graphical abstract

Ocular hypertension induces early hyperactivity in inner retinal inhibitory circuitry before substantial retinal ganglion cell loss. Sustained pharmacological inhibition of GABAergic/glycinergic neurotransmission and Vgat‐targeted Lin28 expression suppress this hyperactivity and improve RGC survival without reducing intraocular pressure. These findings identify presynaptic inhibitory retinal circuits as modifiable targets for glaucoma neuroprotection.

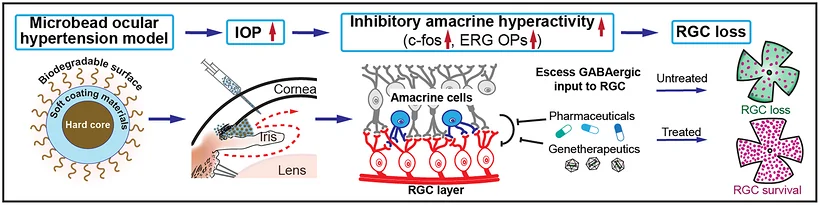

## 1 Introduction

Glaucoma is a neurodegenerative disease defined by progressive loss of retinal ganglion cells (RGCs) and their axons and, in primary open‐angle glaucoma, is most commonly associated with elevated intraocular pressure (IOP) [1]. Reduction of IOP through pharmacological or surgical intervention remains the only clinically validated approach to slow disease progression; however, RGC degeneration frequently persists even when IOP is adequately controlled [2, 3]. This discrepancy points to a key limitation of current therapeutic paradigms and motivates the development of IOP‐independent neuroprotective strategies to preserve RGC structure and function.

To date, most experimental neuroprotective strategies have focused on directly targeting RGCs to delay degeneration and apoptosis [4, 5]. Representative approaches include mitigating excitotoxic stress using NMDA receptor antagonists [6], alpha‐2 adrenergic agonists [7], and calcium channel blockers [8], as well as antioxidant and anti‐inflammatory therapies that mitigate oxidative and inflammatory damage [9]. More recently, stem cell and gene‐based strategies have aimed to enhance intrinsic RGC survival and delay axonal degeneration [10, 11]. In parallel, studies in optic nerve crush models have shown that retinal injury also alters presynaptic retinal circuitry, including amacrine‐cell activity, and that modulation of Lin28 [12, 13], an RNA‐binding regulator of post‐transcriptional gene expression, in amacrine cells can enhance RGC survival and axon regeneration. These studies suggest that non‐cell‐autonomous retinal circuit mechanisms may influence RGC vulnerability after acute axonal injury. Despite encouraging results in preclinical studies, many RGC‐centric interventions face substantial translational barriers, and clinical benefits have remained limited, suggesting that cell‐autonomous protection alone may be insufficient to fully prevent disease progression.

Here, we examine the contribution of presynaptic inhibitory circuitry to glaucomatous neurodegeneration. Different from acute optic nerve crush, ocular hypertension imposes pressure‐driven and temporally progressive stress on the retina, providing a disease context more closely aligned with elevated‐IOP glaucoma. Using mouse models of ocular hypertension, we find that inner retinal neurons exhibit early hyperactivity before detectable RGC loss, accompanied by enhanced oscillatory potential‐associated differential ERG signals. Pharmacological attenuation of inhibitory neurotransmission improved RGC survival, and selective Lin28 expression in Vgat‐positive inhibitory interneurons, achieved via Cre‐dependent AAV delivery, suppressed ocular hypertension‐associated retinal hyperactivity and enhanced RGC preservation (Figure 1A). These findings extend prior injury‐model observations to ocular hypertension‐associated glaucoma and support presynaptic inhibitory circuitry as an early, modifiable determinant of RGC vulnerability rather than a singular upstream driver of disease progression.

**FIGURE 1:**
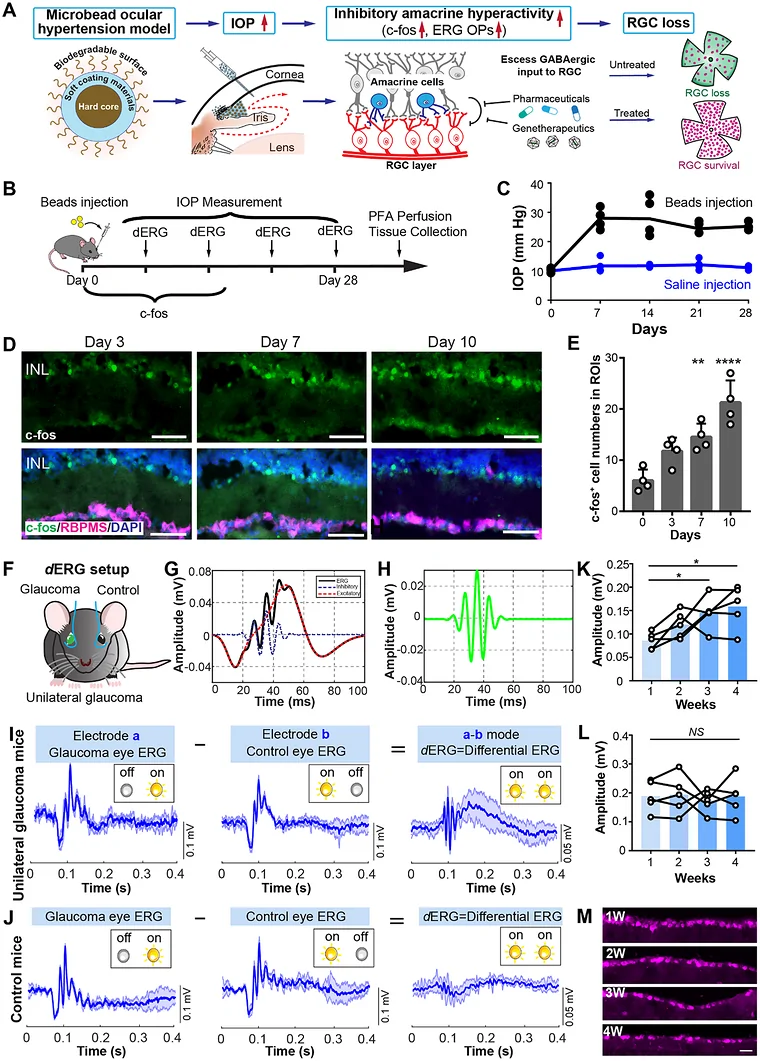
Ocular hypertension induces inner retinal hyperactivity before substantial retinal ganglion cell loss in experimental glaucoma. (A) Schematic of the proposed mechanism and experimental design. In glaucoma, inner retinal inhibitory circuitry becomes hyperactive and is associated with increased RGC vulnerability. Modulation of presynaptic inhibitory circuit activity attenuates retinal hyperactivity and promotes RGC survival. (B) Experimental timeline of the glaucoma mouse model, including injectable viscobeads for intraocular pressure (IOP) elevation and electrophysiological monitoring. (C) IOP measurements over 28 days following viscobeads injection in injected eyes and baseline controls (mean ± standard deviation [S.D.]; n = 5 eyes). (D) Representative confocal images of immunostaining in the inner nuclear layer (INL) of glaucomatous retinas. c‐Fos (green) marks activated cells, RBPMS (magenta) labels RGCs, and DAPI (blue) labels all nuclei. Scale bar, 50 µm. (E) Quantification of c‐Fos‐positive cell numbers in the inner INL over 10 days after viscobead‐induced ocular hypertension (mean ± S.D.; one‐way ANOVA with Tukey's multiple comparisons test, F_(3,12)_ = 17.11; Day 0 vs Day 7, **p = 0.0094; Day 0 vs Day 10, ^****^ *p* < 0.0001; Day 3 vs Day 10, ^**^ *p* = 0.0042; Day 7 vs Day 10, ^*^ *p* = 0.0386; n = 4 eyes per time point). (F) Schematic of the differential electroretinography (*d*ERG) paradigm, in which responses from the glaucomatous eye are subtracted from the contralateral control eye. (G) Diagram of full‐field ERG components, including excitatory inputs from photoreceptors and bipolar cells, and inhibitory feedback from amacrine cells represented by oscillatory potentials (OPs). (H) Isolated high‐frequency OPs associated with inner retinal inhibitory circuit activity. (I–J) Representative scotopic full‐field ERG recordings from individual eyes and corresponding *d*ERG traces from unilateral glaucoma mice (I) and controls (J). (K) Quantification of *d*ERG amplitudes over 4 weeks after induction of ocular hypertension by viscobead injection (mean ± S.D.; one‐way repeated measures ANOVA with Tukey's multiple comparisons test; F_(1.536, 6.146)_ = 7.005; Week 1 vs Week 3, ^*^ *p* = 0.0395; Week 1 vs Week 4, ^*^ *p =* 0.0325; n = 5 mice). (L) Conventional full‐field ERG amplitudes showed non‐significant week‐to‐week variability over 4 weeks after viscobead injection (mean ± S.D.; one‐way repeated measures ANOVA with Tukey's multiple comparisons test; F_(1.560, 6.242)_ = 0.7428; n = 5 mice). (M) Representative fluorescence microscopy images of RBPMS immunostaining in glaucomatous retinas at 4 weeks after induction of ocular hypertension. Scale bar, 50 µm.

## 2 Results

### 2.1 Ocular Hypertension Induces Hyperactivity in Inner Nuclear Layer (INL) Neurons

We first established an experimental glaucoma model by injecting 1 µL of viscobeads, microscale polymeric beads with hybrid mechanical and surface adhesive properties, into the anterior chamber to obstruct aqueous humor outflow and induce ocular hypertension, following our previously validated protocol (see Methods and Figure 1B) [14]. IOP measurements revealed a rapid increase from 10.3 ± 0.6 mmHg before injection (day 0) to 28.0 ± 3.1 mmHg at one week post‐injection (day 7), and IOP remained above 24 mmHg during the following week, confirming stable ocular hypertension in the model (Figure 1C).

To evaluate the effect of glaucomatous stress on retinal cell activity, we quantified c‐fos expression, an immediate early gene marker of neuronal activity [15]. To eliminate environmental light‐driven signals, all mice were dark‐adapted overnight and perfused under red light. In experimental glaucoma mice, c‐fos expression was predominantly localized to the INL (Figure 1D). Quantitative analysis of the inner INL revealed a progressive increase in c‐fos‐positive cell numbers following viscobeads injection, from 6.0 ± 1.9 at baseline (day 0) to 11.8 ± 2.3 on day 3, 14.5 ± 2.3 on day 7, and 21.3 ± 3.8 on day 10 (Figure 1E). This elevated expression persisted throughout the observation period, indicating sustained hyperactivation of INL neurons during ocular hypertension development.

### 2.2 Oscillatory Potentials Reveal Early Retinal Dysfunction in a Unilateral Experimental Glaucoma Model

Electroretinography (ERG) provides a non‐invasive, objective measure of retinal function, but conventional full‐field ERG often lacks sensitivity for detecting early glaucomatous changes [16, 17, 18]. To address this limitation, we developed a differential ERG (*d*ERG) system (Figure 1F) that captures subtle electrophysiological alterations by comparing signals from glaucomatous and control eyes in a unilateral glaucoma model (*d*ERG = ERG_glaucoma_—ERG_control_), providing a sensitive electrophysiological marker for glaucoma. In this setup, one eye was injected intracamerally with viscobeads (“glaucoma” eye), while the contralateral intact eye served as an internal control (“control” eye). Using a differential amplifier in “a‐b” mode, the signal from the glaucomatous eye was subtracted in real time from that of the contralateral control eye. This within‐animal comparison minimized shared background noise and highlighted inter‐eye electrophysiological differences associated with ocular hypertension, enabling sensitive detection of glaucoma‐associated ERG features.

In full‐field ERG recordings [16], retinal responses contain both excitatory and inhibitory components (Figure 1G). Excitatory signals arise primarily driven by photoreceptors and bipolar cells, whereas inhibitory feedback from amacrine cells is reflected in oscillatory potentials (OPs) (Figure 1H). OPs are small wavelets superimposed on the rising phase of the b‐wave and provide a sensitive measure of inner retinal inhibitory circuit activity [16]. Using *d*ERG, we tracked retinal functional changes following viscobeads‐induced ocular hypertension by isolating inter‐eye differences within the same animal. *d*ERG responses revealed prominent OPs‐associated components that progressively increased over four weeks after viscobeads injection (Figure 1I–K), with detectable elevations as early as two weeks after viscobeads delivery (Figure 1I). OP‐associated *d*ERG amplitudes were then quantified from the 30–300 Hz band‐pass‐filtered high‐frequency component of the *d*ERG trace using peak‐to‐peak amplitude within the early post‐flash OP window (see Methods). The increase in OP‐related *d*ERG amplitude reflects enhanced inner retinal circuit activity in the glaucomatous eye relative to the contralateral control eye. In contrast, conventional full‐field ERG recordings obtained from individual eyes showed non‐significant week‐to‐week variability over the same time period (Figure 1L), showing the improved sensitivity of the *d*ERG approach in detecting early functional alterations during glaucoma progression. Comparison with immunohistochemical analyses further showed that OP‐related *d*ERG changes preceded detectable RGC loss by tissue histology (Figure 1M, and Figure S1). Given that OPs predominantly originate from amacrine cell‐mediated inhibitory feedback within the inner retina, these findings suggest that glaucomatous stress induces early hyperactivation of inhibitory interneurons before overt RGC degeneration.

### 2.3 Pharmacological Inhibition of Inhibitory Neurotransmission Promotes RGC Survival in Glaucoma

After observing that ocular hypertension induces hyperactivity in INL neurons (Figure 1D,E and Figure 1K), which are enriched in inhibitory interneurons such as amacrine cells [19]. We next examined whether broad attenuation of inhibitory neurotransmission could influence RGC vulnerability under ocular hypertensive stress. We administered a pharmacological cocktail encapsulated in liposomes designed to block inhibitory synaptic signaling onto RGC dendrites and bipolar cell axon terminals (Figure 2A). This cocktail included bicuculline and strychnine, which antagonize GABA‐A and glycine receptors, respectively, as well as 4‐imidazoleacetic acid, which targets GABAergic inhibition mediated by GABA‐C receptors at amacrine cell synapses [13, 20, 21, 22]. To improve solubility and loading efficiency [23], the drug cocktail was pre‐complexed with cyclodextrin before liposomal encapsulation (DPPC:cholesterol:18:0 TAP). Nanoparticle Tracking Analysis (NTA) and transmission electron microscopy (TEM) results showed that the resulting liposomes had a modal size of 106 nm and a median diameter (D50) of approximately 135 nm (Figure 2B). In vitro release assays with a model hydrophobic compound (Oil Red O) in PBS showed that cumulative drug release increased steadily over four weeks, reaching ∼60% by day 28, consistent with a sustained‐release profile (Figure 2C). Here, these liposomes were used as sustained‐release carriers rather than as cell‐type‐specific delivery vehicles. Given their ∼100–135 nm size and the barrier properties of the inner limiting membrane [24], intact liposomes are expected to remain largely near the vitreous/inner retinal interface while gradually releasing small‐molecule antagonists that diffuse to inner retinal targets.

**FIGURE 2:**
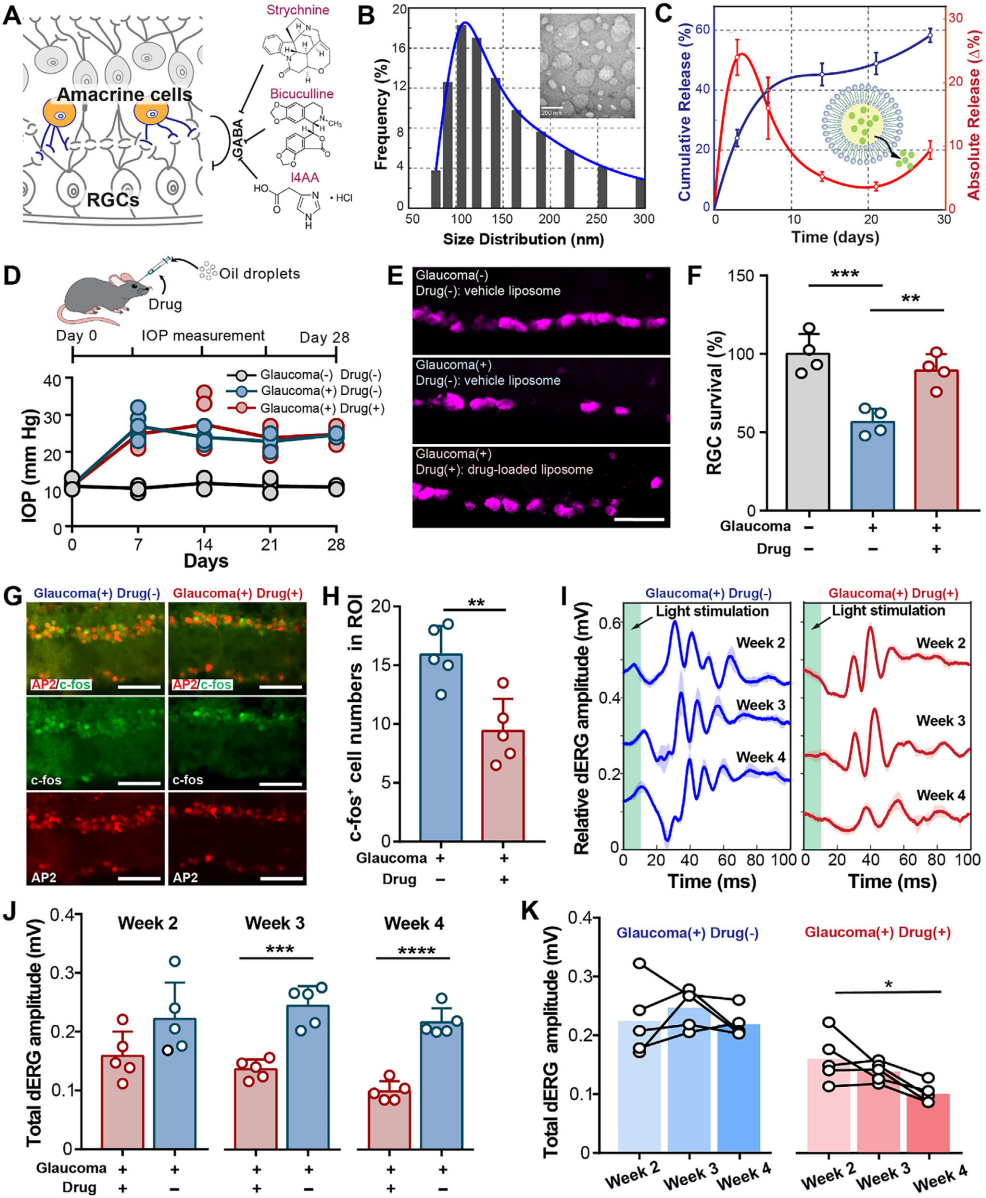
Pharmacological inhibition of inhibitory neurotransmission enhances RGC survival and attenuates retinal circuit hyperactivity during ocular hypertension. (A) Schematic of inhibitory synaptic signaling in the inner retina. Amacrine cells provide GABAergic and glycinergic inputs to RGC dendrites and bipolar cell terminals. The pharmacological cocktail consists of bicuculline (GABA_A_ receptor antagonist), strychnine (glycine receptor antagonist), and 4‐imidazoleacetic acid (I4AA, GABA_C_ receptor antagonist). (B) Characterization of drug‐encapsulated liposomes. Nanoparticle Tracking Analysis (NTA) showed particle size distribution with an inset transmission electron microscopy image of representative spherical vesicles. The modal diameter was 106 nm and D50 was approximately 135 nm. (C) In vitro drug release profile in PBS at 37°C over 4 weeks (n = 4 independent samples). (D) Experimental design for intravitreal administration of liposomes at the time of oil droplets‐induced ocular hypertension and endpoint assessment at 4 weeks (top). IOP measurements over 4 weeks are shown for non‐glaucoma + vehicle (non‐glaucoma mice treated with vehicle liposomes without drug loading), glaucoma + vehicle (glaucoma mice treated with vehicle liposomes without drug loading), and glaucoma + drug (glaucoma mice treated with drug‐loaded liposomes) (bottom; n = 5 eyes). (E) Representative fluorescence microscopy images of retinal flat‐mount RGCs, labeled with RBPMS (magenta), from non‐glaucoma + vehicle, glaucoma + vehicle, and glaucoma + drug. Scale bar, 50 µm. (F) Quantification of RGC density corresponding to (E) (mean ± S.D.; one‐way ANOVA with Tukey's multiple‐comparisons test; F(2,9) = 18.04; non‐glaucoma + vehicle versus glaucoma + vehicle, ^***^ *p* = 0.0007; glaucoma + vehicle versus glaucoma + drug, ^**^ *p* = 0.0047). (G) Representative c‐Fos immunofluorescence microscopy images in the inner nuclear layer from glaucoma + vehicle and glaucoma + drug. c‐Fos (green) marks activated cells and AP2 (red) labels amacrine cells. Scale bar, 50 µm. (H) Quantification of c‐Fos‐positive cell number in the inner nuclear layer corresponding to (G) (mean ± S.D.; unpaired t test; ^**^ *p* = 0.0042). (I) Representative *d*ERG traces at weeks 2, 3, and 4 after induction of ocular hypertension from glaucoma + vehicle and glaucoma + drug. (J) Quantification of oscillatory potential‐associated *d*ERG amplitudes at weeks 2 to 4 corresponding to (I) (mean ± S.D.; unpaired two‐tailed t‐test; week 2, *p* = 0.0935; week 3, ^***^ *p* = 0.0002; Mann‐Whitney test; week 4, ^**^ *p* = 0.0079). (K) Longitudinal quantification of oscillatory potential‐associated *d*ERG amplitudes across weeks 2 to 4 in glaucoma + vehicle and glaucoma + drug (mean ± S.D.; one‐way ANOVA with Tukey's multiple‐comparisons test; F_(1.2, 4.8)_ = 7.958, week 2 versus week 4, ^*^ *p*= 0.0111; n = 5 mice).

Given the approximately one‐month release profile of the liposomal formulation, the viscobeads‐injection model did not induce sufficient RGC loss within this time frame (Figure S1). We therefore adopted a silicone oil droplet‐induced ocular hypertension model [25], optimized from our previous work [23], to achieve sufficient RGC degeneration over a shorter period. For in vivo experiments, we intravitreally injected liposomal formulations on the same day as silicone oil droplet delivery in wild‐type mice to ensure sustained inhibition of inhibitory neurotransmission (Figure 2D, top). Over the four‐week evaluation period after silicone oil droplet injection, we observed rapid IOP elevations. Administration of the pharmacological compounds did not alter IOP compared with vehicle‐loaded liposomes (24.8 ± 1.9 mmHg vs. 24.6 ± 0.5 mmHg in glaucomatous eyes). In contrast, saline‐injected control mice without oil droplet injection exhibited an IOP of 10.6 ± 0.5 mmHg at 28 days post‐injection (Figure 2D, bottom).

At four weeks post‐injection, we examined the retinal flat mounts by immunostaining and found that RGC survival was higher in glaucoma mice treated with the inhibitory receptor antagonist cocktail (89.4 ± 10.5%, glaucoma + drug) compared with glaucoma mice treated with vehicle liposomes without drug loading (56.2 ± 8.4%, glaucoma + vehicle) (Figure 2E,F). Interestingly, pharmacological blockade of inhibitory neurotransmission reduced c‐fos expression in INL amacrine cells, particularly in AP2α‐positive interneurons, at 7 days post‐injection (Figure 2G,H). While the drug cocktail was designed to block inhibitory inputs onto RGCs, components such as the GABA‐C receptor antagonist may also act at amacrine terminals, and network‐level disinhibition may dampen hyperactive firing across the inhibitory network [26, 27], providing a potential explanation for the observed decrease in c‐fos (Figure 2G,H).

Taking advantage of the sensitivity of *d*ERG for detecting early glaucomatous progression, we deployed *d*ERG to monitor retinal electrophysiological changes with or without pharmacological treatment (Figure 2I–K). Consistent with the c‐fos results, OP‐associated amplitudes (total *d*ERG amplitude) in vehicle‐treated glaucomatous eyes (glaucoma + vehicle) increased by week 2 (0.22 ± 0.06 mV) and remained elevated through weeks 3 and 4 (0.24 ± 0.03 and 0.22 ± 0.02 mV, respectively). In antagonist‐treated eyes (glaucoma + drug), OP‐associated amplitudes progressively declined from week 2 to week 4 (0.16 ± 0.04, 0.14 ± 0.02, and 0.10 ± 0.02 mV, respectively), reflecting reduced inner retinal circuit hyperactivity. These findings suggest that sustained broad blockade of inhibitory neurotransmission attenuates ocular hypertension‐associated inner retinal hyperactivity and improves RGC survival.

### 2.4 Genetic Manipulation of Lin28 Expression in Inhibitory Interneurons Mediates Circuit‐Level Neuroprotection in Experimental Glaucoma

Prior work in optic nerve crush models showed that Lin28 expression in amacrine cells can suppress injury‐associated retinal hyperactivity and enhance RGC survival and axon regeneration [13]. Building on these studies, we next asked whether Lin28‐mediated modulation of presynaptic retinal circuitry could also influence RGC vulnerability in ocular hypertension‐associated glaucoma, a pressure‐driven and temporally progressive disease model distinct from acute axonal injury. Lin28 is an RNA‐binding regulator of post‐transcriptional gene expression and has been implicated in neuronal injury responses and growth‐factor responsiveness. Because Lin28 has pleiotropic molecular functions, we used it here as a genetic strategy to modulate retinal circuit state rather than as evidence for a single defined downstream pathway.

To investigate whether Lin28 expression in inhibitory retinal interneurons improves RGC survival during ocular hypertension, we delivered Cre‐dependent AAV2‐FLEX‐Lin28 to Vgat‐Cre mice to target Vgat‐positive inhibitory interneurons. As a comparison group, we delivered the same Cre‐dependent Lin28 virus to Vglut2‐Cre mice. After two weeks of incubation, we injected viscobeads to the anterior chamber to induce ocular hypertension (Figure 3A). In Vglut2‐Cre mice, Lin28 expression was directed to Vglut2‐lineage excitatory retinal neurons, including a substantial population of RGCs, whereas in Vgat‐Cre mice, Lin28 was expressed in Vgat‐positive inhibitory interneurons of the inner retina, including amacrine cells [28, 29]. Fluorescence microscopy confirmed successful Lin28 expression in distinct retinal cell populations (Figure 3B–C).

**FIGURE 3:**
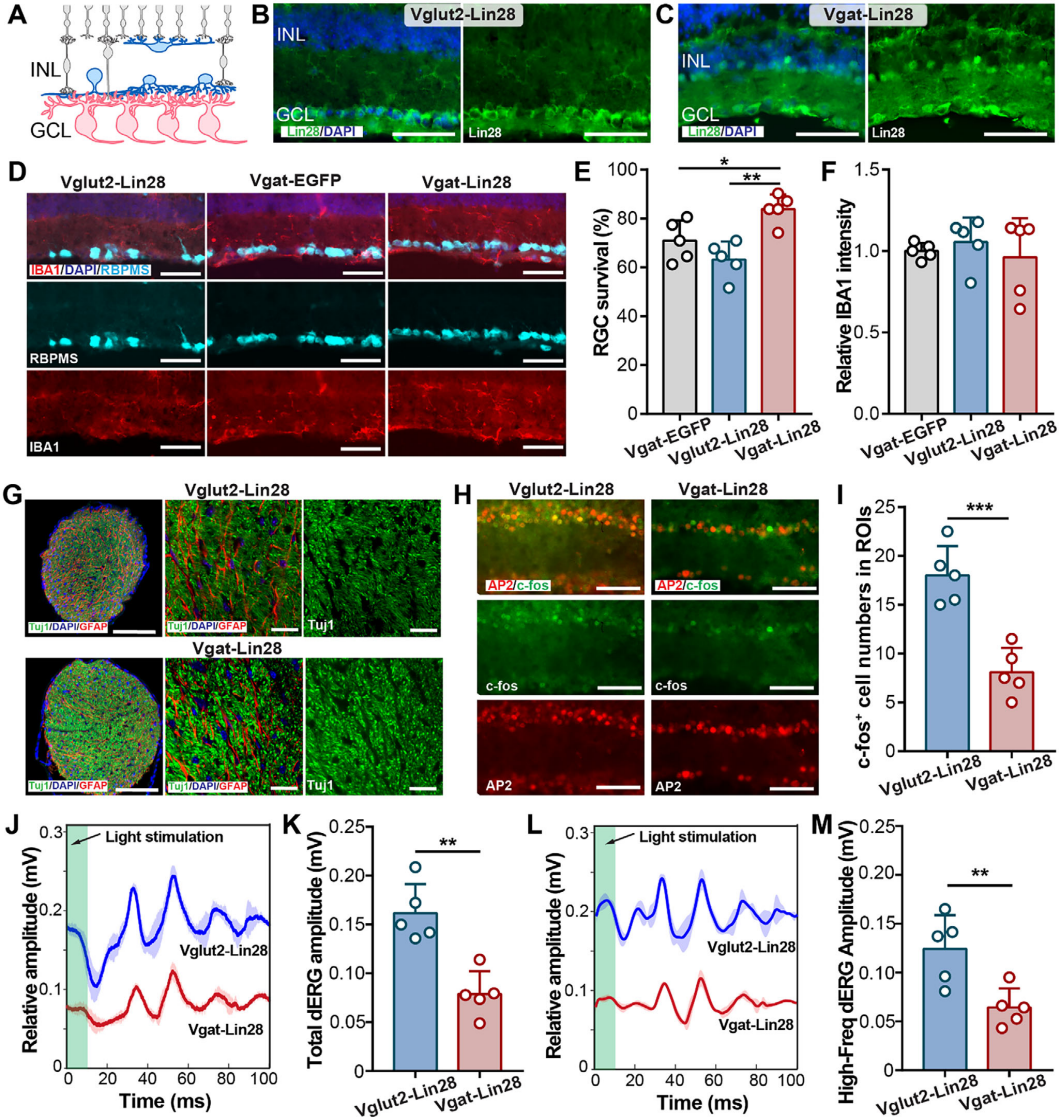
Selective Lin28 expression in inhibitory interneurons suppresses retinal hyperactivity and preserves RGCs in glaucoma. (A) Strategy for Cre‐dependent Lin28 expression using AAV2‐FLEX‐Lin28 in Vglut2‐Cre mice to target Vglut2‐lineage excitatory retinal neurons, including RGCs, and in Vgat‐Cre mice to target inhibitory interneurons, including amacrine cells. INL, inner nuclear layer; GCL, ganglion cell layer. (B,C) Representative immunofluorescence images validating Lin28 expression after AAV‐FLEX‐Lin28 delivery in retinas of Vglut2‐Cre and Vgat‐Cre mice. Lin28 (green) labels expressing cells, and DAPI (blue) labels all nuclei. Scale bar, 50 µm. (D) Representative immunofluorescence images of retinal flat mounts from Vglut2‐Lin28, Vgat‐Cre mice injected with control virus AAV2‐FLEX‐EGFP (Vgat‐EGFP), and Vgat‐Lin28 groups stained for RBPMS (cyan) to label RGCs, IBA1 (red) to label microglia, and DAPI (blue) to label all nuclei. Scale bar, 50 µm. (E) Quantification of RGC survival, calculated as the number of RGCs relative to the total number of DAPI‐positive cells within the region of interest (ROI), across groups at 8 weeks after viscobeads‐induced ocular hypertension (mean ± S.D.; one‐way ANOVA with Tukey's multiple‐comparisons test; n = 5 eyes; F_(2,12)_ = 10.25; Vgat‐EGFP versus Vglut2‐Lin28, P = 0.2522; Vgat‐EGFP versus Vgat‐Lin28, ^*^ *p* = 0.0396; Vglut2‐Lin28 versus Vgat‐Lin28, ^**^ *p* = 0.0020). (F) Quantification of IBA1 immunoreactivity across groups at 8 weeks after viscobeads‐induced ocular hypertension (mean ± S.D.; Kruskal‐Wallis ANOVA; *p* = 0.4441). (G) Representative immunofluorescence images of optic nerve cross‐sections stained for Tuj1 (green) to label all neuronal cells and GFAP (red) to label astrocytes from Vglut2‐Lin28 (top) and Vgat‐Lin28 (bottom) mice. Scale bars, 100 µm (left) and 20 µm (middle and right). (H) Representative c‐Fos immunofluorescence images in the inner nuclear layer from Vglut2‐Lin28 and Vgat‐Lin28 retinas. c‐Fos (green) marks activated cells and AP2 (red) labels amacrine cells. Scale bar, 50 µm. (I) Quantification of c‐Fos‐positive cell number in the inner nuclear layer corresponding to (H) (mean ± S.D.; unpaired t test; ^***^ *p* = 0.0005). (J) Representative *d*ERG traces from Vglut2‐Lin28 and Vgat‐Lin28 mice. (K) Quantification of total inter‐eye differential amplitudes derived from *d*ERG recordings corresponding to (J) (mean ± S.D.; unpaired two‐tailed t test; ^**^ *p* = 0.0012). (L) Isolated high‐frequency components of *d*ERG responses corresponding to OPs. (M) Quantification of OP‐associated *d*ERG amplitudes corresponding to (L) (mean ± S.D.; unpaired two‐tailed t test; ^**^ *p* = 0.0098).

Eight weeks after induction of ocular hypertension, we observed enhanced RGC survival in Vgat‐Lin28 mice, which express Lin28 in inhibitory interneurons (83.9 ± 6.0%), compared with Vgat‐Cre mice receiving the control virus AAV2‐FLEX‐EGFP (Vgat‐EGFP, 71.0 ± 8.2%) (Figure 3D,E). In contrast, Vglut2‐Lin28 mice, in which Lin28 was expressed in Vglut2‐lineage excitatory retinal neurons including RGCs, exhibited a lower survival rate (63.2 ± 7.4%), suggesting that modulation of presynaptic inhibitory circuitry mediated neuroprotection. We next examined whether Lin28‐mediated protection was associated with changes in retinal glial activation. Neither Vgat‐Lin28 nor Vglut2‐Lin28 substantially altered IBA1‐positive microglial activation or GFAP‐positive reactive gliosis during glaucomatous progression compared with the corresponding glaucoma control condition (Figure 3F and Figure S2). These results suggest that the protective effect of Vgat‐targeted Lin28 is unlikely to arise from broad suppression of microglial or astroglial activation. Instead, Lin28‐mediated neuroprotection appears to occur despite persistent glial activation, although more subtle changes in inflammatory signaling, oxidative stress, or glial functional states may still contribute to retinal circuit dysfunction and RGC vulnerability. Upon further assessment of optic nerve integrity, we also found enhanced axonal preservation in Vgat‐Lin28 mice compared with Vglut2‐Lin28 mice (Figure 3G), indicating that interneuron‐targeted modulation preserves both somatic and axonal compartments of RGCs.

To test the hypothesis that inhibitory amacrine cell activity contributes to the observed neuroprotective effects during ocular hypertension, we further examined amacrine cell activity by immunohistological staining for c‐fos together with AP2α labeling. c‐fos expression in AP2α‐labeled amacrine cells was lower in Vgat‐Lin28 mice than in Vglut2‐Lin28 mice (Figure 3H‐I, and Figure S3A). Electrophysiological assessment by *d*ERG further showed reduced total inter‐eye differential amplitudes (Figure 3J‐K, and Figure S3B) and reduced high‐frequency components of the *d*ERG response (Figure 3L,M) in Vgat‐Lin28 mice relative to Vglut2‐Lin28 mice. These results indicate that Lin28 expression in inhibitory interneurons suppresses ocular hypertension‐induced circuit hyperactivity, supporting a presynaptic mechanism underlying neuroprotection.

Amacrine cells provide inhibitory synaptic input to RGC dendrites and regulate retinal output gain [30]. Previous work demonstrated that ONC injury induces pathological amacrine cell hyperactivity that suppresses RGC function and survival [13, 31]. Our findings extend this circuit‐level principle to experimental glaucoma by showing that ocular hypertension is associated with early and sustained enhancement of inner retinal inhibitory circuit activity. These data support a model in which altered presynaptic inhibitory circuitry contributes to RGC vulnerability during glaucomatous stress, rather than acting as a singular upstream driver of degeneration. Targeting presynaptic inhibitory circuitry, in combination with RGC‐directed approaches, may therefore represent a mechanistically distinct and complementary strategy for neuroprotection in glaucoma.

### 2.5 Lin28 Expression in Inhibitory Interneurons Mitigates Sleep Alterations Associated with Ocular Hypertension

With cellular and circuit‐level evidence that suppression of inhibitory interneuron hyperactivity promotes RGC survival in experimental glaucoma, we next examined whether ocular hypertension‐associated retinal circuit modulation was associated with changes in sleep architecture. Given that sleep disturbances have been reported in patients with glaucoma [32, 33], we next asked whether modulation of inner retinal inhibitory circuitry influences sleep behavior during ocular hypertension (Figure 4A).

**FIGURE 4:**
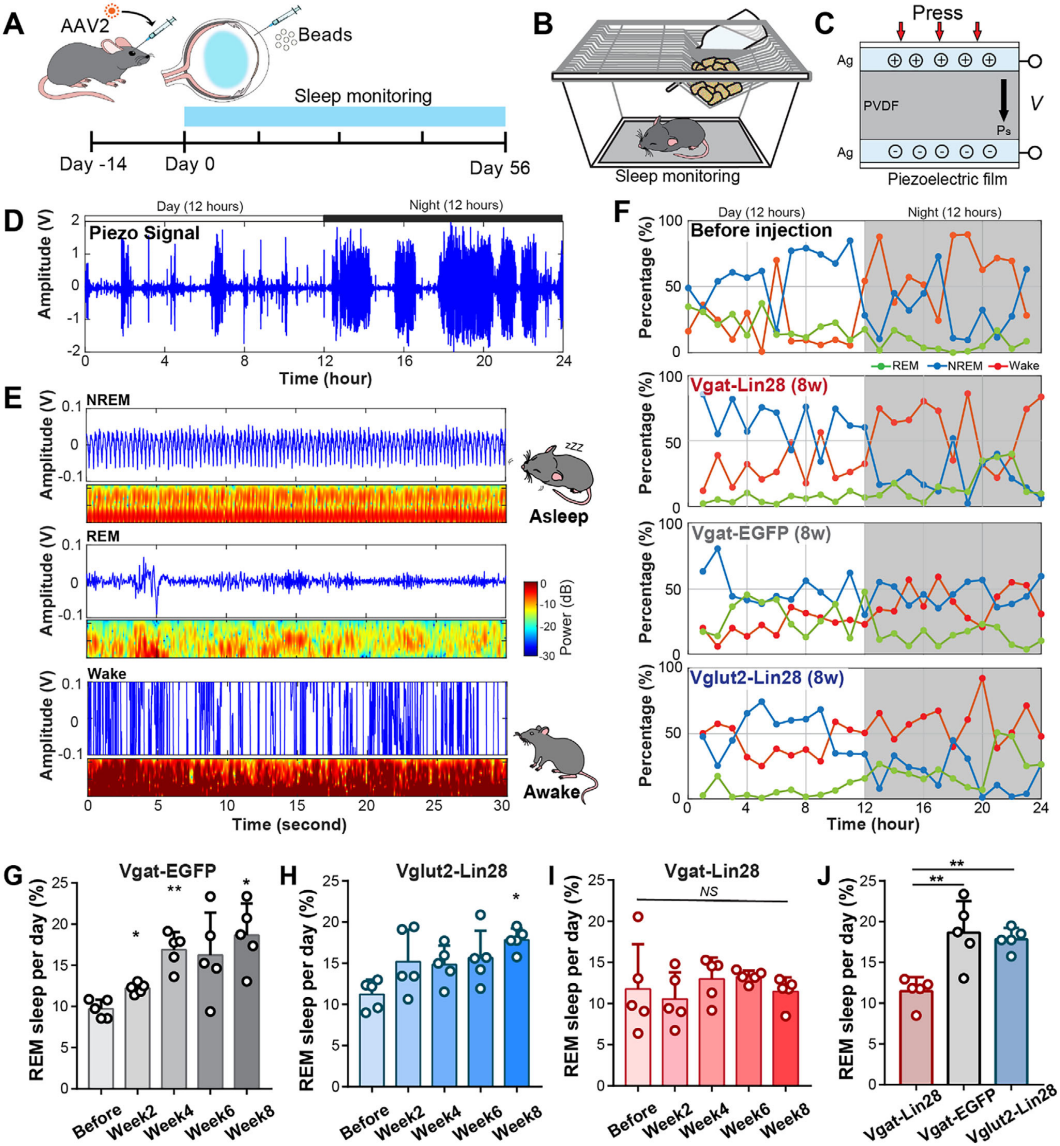
Lin28 expression in inhibitory interneurons is associated with mitigation of sleep alterations during ocular hypertension. (A) Experimental design showing intravitreal delivery of AAV2‐FLEX‐Lin28 2 weeks before induction of ocular hypertension by viscobeads injection, followed by longitudinal sleep monitoring. (B,C) Schematic of the noninvasive PiezoSleep system. Piezoelectric sensors in (C) positioned beneath the cage floor detect respiration‐ and movement‐related pressure changes in freely behaving mice under a 12‐h light/12‐h dark cycle. (D) Representative 24‐h PiezoSleep recording from a Vgat‐Lin28 mouse across one light‐dark cycle, showing wakefulness associated with high‐amplitude signals and sleep states associated with reduced amplitude. (E) Expanded PiezoSleep traces showing classification of behavioral states based on signal amplitude and spectral features, including wakefulness, rapid eye movement (REM) sleep with behavioral quiescence and irregular respiration, and non‐rapid eye movement (NREM) sleep with quiescence and regular respiration. Corresponding piezo signal amplitude (top) and spectrogram (0 to 20 Hz, bottom) are shown. (F) Sleep percentages of wake, REM, and NREM states before viscobeads injection and at 8 weeks after induction of ocular hypertension by viscobeads injection in Vgat‐Lin28 mice, Vgat‐EGFP mice, and Vglut2‐Lin28 mice. Each dot represents the average value within a 30‐min bin. (G) REM sleep proportion in Vgat‐EGFP mice before and at 2, 4, and 8 weeks after induction of ocular hypertension (mean ± S.D.; one‐way ANOVA with Tukey's multiple‐comparisons test; n = 5 mice; F_(1.322,5.286)_ = 10.15; versus before injection, week 2, ^*^ *p* = 0.0464; week 4, ^**^ *p* = 0.0033; week 8, ^*^ *p* = 0.0107). (H) REM sleep proportion in Vglut2‐Lin28 mice before and at 2, 4, and 8 weeks after induction of ocular hypertension (mean ± S.D.; one‐way ANOVA with Tukey's multiple‐comparisons test; n = 5 mice; F_(2.097,8.389)_ = 5.144; versus before injection, week 8, ^*^ *p* = 0.0315). (I) REM sleep proportion in Vgat‐Lin28 mice before and at 2, 4, and 8 weeks after induction of ocular hypertension (mean ± S.D.; one‐way ANOVA with Tukey's multiple‐comparisons test; n = 5 mice; F_(1.588,6.354)_ = 0.7855; *p* = 0.4674). (J) REM sleep percentage among Vgat‐EGFP, Vgat‐Lin28, and Vglut2‐Lin28 mice at 8 weeks after induction of ocular hypertension (mean ± S.D.; one‐way ANOVA with Tukey's multiple‐comparisons test; F_(2,12)_ = 11.75; Vgat‐Lin28 versus Vgat‐EGFP, ^**^ *p* = 0.0022; Vgat‐Lin28 versus Vglut2‐Lin28, ^**^ *p* = 0.0052).

We deployed a fully noninvasive platform for longitudinal monitoring of sleep and circadian behavior in freely moving mice (Figure 4B,C). This system uses piezoelectric force‐pad sensors positioned beneath the cage floor to detect respiration‐ and movement‐related pressure fluctuations, coupled to high‐resolution data acquisition for continuous recording [34, 35]. Sleep‐wake states are classified based on motion (Teager energy, TE), breathing rate, and breathing rate variability (BRV): wakefulness is characterized by high TE; rapid eye movement (REM) sleep by behavioral quiescence with irregular respiration; and non‐REM (NREM) sleep by quiescence with regular respiration (Figure 4D,E), as also described in other studies [34, 35]. Low‐frequency band‐pass filtering (0.5–18 Hz) minimizes environmental noise and movement artifacts. This approach enables extended monitoring without the surgical implantation and tethering required for conventional EEG/EMG recordings, thereby preserving naturalistic behavior.

For sleep behavior assessment, we injected AAV2‐FLEX‐Lin28 intravitreally to tested mice two weeks before viscobeads injection for induction of ocular hypertension. Mice were recorded continuously for 36 h, and data were normalized to a 24‐h period spanning a 12‐h light/dark cycle (Figure 4D). The traces were then classified into wake, REM, and NREM states (see Methods, Figure 4E). Before induction of ocular hypertension, mice displayed normal circadian sleep architecture, with predominant sleep during the light phase and increased wakefulness during the dark phase (Figure 4F, and Figure S4). Following viscobead injection to induce ocular hypertension, sleep patterns diverged across groups, particularly with respect to REM sleep. In Vgat‐EGFP control mice, the REM sleep percentage increased over eight weeks, from 11.2 ± 1.8% at baseline to 17.8 ± 1.4% (Figure 4G). A similar increase was observed in Vglut2‐Lin28 mice, rising from 9.7 ± 1.1% at baseline to 18.7 ± 3.9% eight weeks post‐injection (Figure 4H). However, Vgat‐Lin28 mice showed no significant change in REM sleep following ocular hypertension (11.8 ± 5.4% at baseline vs. 11.5 ± 1.7% at eight weeks post‐injection) (Figure 4I). Direct comparison at eight weeks revealed higher REM sleep percentage in Vgat‐EGFP and Vglut2‐Lin28 mice compared with Vgat‐Lin28 mice (Figure 4J).

These results indicate that ocular hypertension is associated with altered sleep patterns, particularly increased REM sleep. Selective Lin28 expression in inhibitory interneurons (Vgat‐Lin28), but not in Vglut2‐lineage excitatory retinal neurons (Vglut2‐Lin28), was associated with mitigation of ocular hypertension‐associated sleep alterations. Given that Vgat‐targeted Lin28 expression suppresses inner retinal hyperactivity, as evidenced by reduced c‐fos expression (Figure 3H,I) and diminished OPs in *d*ERG recordings (Figure 3J–M), and preserves RGC survival (Figure 3E), the mitigation of REM sleep disturbance in experimental glaucoma suggests an association between retinal inhibitory circuit dysfunction and broader sleep‐related physiological changes during glaucoma progression. These results suggest that ocular hypertension‐associated retinal circuit dysfunction may be accompanied by broader physiological changes in sleep architecture. Vgat‐targeted Lin28 expression mitigated both retinal hyperactivity and sleep alterations, supporting the physiological relevance of inhibitory retinal circuit modulation while highlighting the need for future studies to define the specific retinal and central pathways linking ocular hypertension to sleep disruption.

## 3 Discussion and Conclusion

Overall, in this study, we demonstrate that ocular hypertension is associated with early hyperactivation of inner retinal circuitry before substantial RGC loss is detected, as evidenced by increased c‐fos expression in the inner nuclear layer and enhanced oscillatory potential‐associated components detected by differential ERG. Pharmacological blockade of inhibitory neurotransmission improved RGC survival without altering intraocular pressure, supporting a functional contribution of presynaptic inhibitory signaling to RGC vulnerability under ocular hypertensive stress. Genetic modulation using Cre‐dependent Lin28 expression further showed that targeting Vgat‐positive inhibitory interneurons suppressed inner retinal hyperactivity, reduced OP‐associated dERG abnormalities, and enhanced RGC survival more effectively than Lin28 expression in Vglut2‐lineage excitatory retinal neurons. In addition, ocular hypertension was associated with altered sleep architecture, and Vgat‐targeted Lin28 expression was associated with mitigation of these sleep alterations. Together, these findings identify presynaptic inhibitory retinal circuitry as an early and modifiable contributor to RGC vulnerability in experimental glaucoma, while avoiding the interpretation that inhibitory circuit dysfunction is a singular upstream driver of disease progression.

Our findings extend previous optic nerve crush studies demonstrating that injury‐induced amacrine cell hyperactivity can impair RGC function and survival, and that enhancing Lin28 expression in amacrine cells promotes RGC survival and axon regeneration. We expand this circuit‐level concept to ocular hypertension‐induced glaucoma, a chronic neurodegenerative condition characterized by progressive pressure‐dependent retinal stress rather than acute axonal injury. These results suggest that inhibitory retinal circuits may represent an important regulatory interface between IOP elevation, glial activation, metabolic stress, and intrinsic RGC vulnerability during glaucoma progression.

In the meantime, several limitations should be noted. First, although c‐fos activation and OP‐associated *d*ERG changes indicate altered inner retinal activity, they do not directly define inhibitory synaptic remodeling onto RGCs; future electrophysiological studies, including patch‐clamp recordings of inhibitory postsynaptic currents, are needed to establish the underlying circuit mechanisms. Second, the pharmacological rescue approach broadly targeted GABAergic and glycinergic signaling, which may disrupt normal retinal processing, and was evaluated in a different glaucoma model than the genetic experiments; receptor‐specific studies with improved targeting or temporal control, together with cross‐model validation, will be needed to define pathway contributions, safety, and generalizability. Third, although Vglut2‐Cre and Vgat‐Cre strategies revealed distinct effects of Lin28 modulation in excitatory and inhibitory neurons, further cell‐type‐specific and molecular analyses are required to identify the relevant interneuron subtypes and downstream mechanisms. Fourth, the AAV experiments used prophylactic viral delivery before ocular hypertension induction, which does not reflect typical clinical intervention after disease onset or risk identification, and the long‐term safety of Lin28 overexpression requires further evaluation. Finally, while our findings suggest an association between ocular hypertension, retinal circuit dysfunction, and altered sleep architecture, future studies are needed to directly determine the contribution of melanopsin‐expressing intrinsically photosensitive RGCs, optic nerve signaling, and central circadian pathways to these behavioral changes.

## 4 Materials and Methods

### 4.1 Liposome Preparation and Characterization

1,2‐Dipalmitoyl‐sn‐glycero‐3‐phosphocholine (DPPC; 850355) and 1,2‐stearoyl‐3‐trimethylammonium‐propane chloride (18:0 TAP; 890880P) were obtained from Avanti Polar Lipids, cholesterol (AAA1147018) from Thermo Fisher Scientific, and strychnine (S0532), (+)‐bicuculline (14340), and 4‐imidazoleacetic acid hydrochloride (I4AA; 219991) from Sigma–Aldrich. Lipids were dissolved in chloroform at 10 mg mL^−^^1^ and combined at a volumetric ratio of 1:2:1 (DPPC:cholesterol:18:0 TAP). To enhance aqueous compatibility, (+)‐bicuculline, I4AA, and strychnine were first incorporated into cyclodextrin inclusion complexes (2‐hydroxypropyl‐β‐cyclodextrin (HPβCD)) (20 mg bicuculline, 15 mg I4AA, and 2 mg strychnine in 2 mL cyclodextrin solution). The drug‐cyclodextrin complex was mixed with the lipid phase to form a biphasic system and sonicated using a Qsonica Q55 sonicator at 15% amplitude for 30 s twice to promote emulsification and encapsulation. Poloxamer F‐68 was added to a final concentration of 0.25% to stabilize the suspension. Chloroform was removed under reduced pressure with intermittent vortexing. The suspension was centrifuged at 10 000 g for 10 min, and the resulting pellet was resuspended in PBS.

Drug release was evaluated using Oil Red O as a model hydrophobic compound. Liposomes were incubated in PBS at 37°C. At designated time points, samples were centrifuged, supernatants were collected for absorbance measurement by UV–vis spectroscopy, and pellets were resuspended in fresh PBS. Cumulative release was calculated based on a standard calibration curve.

Liposome morphology and size were assessed by transmission electron microscopy (TEM; JEOL 2100F). Suspensions were deposited onto carbon‐coated copper grids, negatively stained, and examined to confirm vesicular structure.

### 4.2 Animals

All animal procedures were approved by the Institutional Animal Care and Use Committee at Binghamton University (Protocol 894‐23) and conducted in accordance with institutional and NIH guidelines. Mice were maintained under a 12 h light and 12 h dark cycle with access to food and water. Male and female C57BL/6J (stock no. 000664), Vglut2‐Cre (stock no. 028863), and Vgat‐Cre (stock no. 028862) mice, aged 6 to 8 weeks, were obtained from The Jackson Laboratory and used for experiments.

### 4.3 Intracameral Injection

Mice were anesthetized by intraperitoneal administration of ketamine (100 mg kg^−^^1^) and xylazine (10 mg kg^−^^1^). Under a surgical microscope, the central cornea was carefully penetrated with a 33‐gauge needle (CAD4113, Sigma). A small air bubble was introduced through the incision to minimize reflux from the anterior chamber. Viscobeads (1 µL) were then delivered into the anterior chamber via the same entry site. Following a 5 min period to permit bead redistribution toward the iridocorneal angle, antibiotic ophthalmic ointment (Vetropolycin, Dechra Veterinary Products) was applied. Animals were maintained on a heating pad until full recovery from anesthesia.

### 4.4 AAV Vector Delivery and Validation

Cre‐dependent AAV vectors were provided by the Boston Children's Hospital Viral Core. The vectors included pAAV‐CAG‐FLEX‐GFP as the control construct and pAAV‐CAG‐FLEX‐Lin28a for Lin28a expression. Both constructs used a CAG promoter and a Cre‐dependent double‐floxed inverted open reading frame to restrict transgene expression to Cre‐positive cells. Viral preparations were used at titers of at least 1.0 × 10^1^ ^3^ GC mL^−^^1^. For intravitreal delivery, 2 µL of virus was injected into Vgat‐Cre or Vglut2‐Cre mice using a glass‐pulled micropipette inserted near the ora serrata. A small volume of aqueous humor was withdrawn before injection to minimize acute IOP elevation. Viscobead injection was performed two weeks after AAV delivery to allow transgene expression before ocular hypertension induction. Transgene expression was validated by GFP or Lin28a immunofluorescence in retinal sections.

### 4.5 Experimental Timeline

For pharmacological inhibition experiments, drug‐loaded or vehicle liposomes were intravitreally injected, and silicone oil droplets were intracamerally injected into the same eye on the same day to induce ocular hypertension. IOP and retinal outcomes were assessed at the indicated time points.

For genetic manipulation experiments, AAV2‐FLEX‐Lin28a or AAV2‐FLEX‐GFP was intravitreally injected into Vgat‐Cre or Vglut2‐Cre mice. After two weeks of viral expression, ocular hypertension was induced by intracameral viscobead injection, followed by IOP monitoring, dERG recording, and endpoint histological analysis.

### 4.6 Intraocular Pressure (IOP) Measurement

IOP was assessed using a TonoLab rebound tonometer (iCare, Colonial Medical Supply). Mice were lightly anesthetized with 1.0 to 1.5% isoflurane delivered in 100% oxygen during measurement. For each eye, the instrument obtained five consecutive readings per cycle, automatically discarding the highest and lowest values and calculating a mean from the remaining measurements. Three independent cycles were performed per eye, and the resulting values were averaged to obtain the final IOP.

### 4.7 Differential Electroretinogram (*d*ERG) Recording

The *d*ERG was recorded bilaterally using a custom‐built programmable acquisition system. Mice were dark‐adapted before recording and maintained in darkness or under dim red illumination throughout the procedure. Anesthesia was induced by intraperitoneal administration of ketamine (100 mg·kg^−^^1^) and xylazine (10 mg·kg^−^^1^), and pupils were dilated with 1% tropicamide. Coiled silver wire electrodes (0.5 mm diameter; Sigma–Aldrich, Cat# 265586) were gently positioned on the corneal surface. Signals were amplified (10 000×) using a DAM50 extracellular amplifier, band‐pass filtered between 1 and 1000 Hz, and digitized at 10 000 Hz with a PowerLab 8/35 acquisition system (ADInstruments). Flash stimuli consisting of five 10 ms pulses delivered at 0.2 Hz were presented to both eyes via the PowerLab 8/35 trigger output, and responses were recorded for subsequent analysis.

For *d*ERG analysis, total *d*ERG amplitude was quantified as the peak‐to‐peak amplitude within the post‐stimulus response window after 60 Hz notch filtering. OP‐associated *d*ERG amplitude was extracted from the high‐frequency component of the *d*ERG trace after band‐pass filtering at 30–300 Hz with 60 Hz notch filtering, and peak‐to‐peak amplitudes were quantified within the early post‐flash window corresponding to OP wavelets. The same analysis parameters were applied across groups and time points.

### 4.8 Sleep Recording and Analysis

Sleep‐wake behavior was assessed using the PiezoSleep system (Signal Solutions, LLC), which consists of individually housed cages equipped with piezoelectric sensors under an automated 12 h light and 12 h dark cycle. Mice were recorded continuously for 36 h, and data were normalized to a 24‐h period spanning a 12‐h light/dark cycle. This platform has been validated against EEG and EMG recordings, demonstrating approximately 92% agreement in sleep‐wake classification. Signal traces were processed using a custom MATLAB‐based pipeline. Data were filtered with a band‐pass filter to minimize noise and movement artifacts, retaining frequencies between 0.5 and 18 Hz relevant to respiratory and motion‐related signals. Spectral features were extracted using Fast Fourier Transform analysis. Behavioral states were classified in 2 s epochs as wakefulness or sleep based on the root mean square power within the 2 to 4 Hz band, consistent with established methods. REM and NREM sleep were classified based on respiratory regularity and spectral features, with REM sleep defined by behavioral quiescence and irregular respiration and NREM sleep defined by behavioral quiescence and regular respiration.

### 4.9 Immunohistochemistry

Animals were deeply anesthetized and euthanized, followed by transcardial perfusion with ice‐cold phosphate‐buffered saline (PBS) and then 4% paraformaldehyde (PFA; Sigma). Optic nerves were isolated and immersed in 4% PFA at 4°C overnight for postfixation. Tissues were cryoprotected in 30% sucrose in PBS for 48 h, embedded in Optimal Cutting Temperature compound (Tissue‐Tek), and frozen on dry ice. Optic nerve sections were cut at 12 µm thickness, while retinal sections were cut at 12 µm thickness. Retinal and optic nerve cryosections were washed in PBS and incubated for 30 min at room temperature in blocking solution containing PBS, 1% Triton X‐100, and 5% horse serum. Primary antibodies (Table 1) diluted in the same blocking buffer were applied and incubated overnight at room temperature. After three 10 min washes in PBS, sections were incubated with species‐appropriate secondary antibodies diluted 1:1000 in PBS for 2 h at room temperature. Samples were then washed five times in PBS, mounted with Fluoromount‐G (Southern Biotech), and imaged. Primary antibodies are listed below. For RGC quantification, retinal sections containing the optic nerve head were selected to ensure comparable anatomical sampling. The RGC layer was manually defined as the region of interest, and RBPMS‐positive cells were counted using Cell Count software followed by manual verification. For flat‐mount analyses, equivalent retinal regions were imaged across groups using identical acquisition settings, and RGC density or survival percentage was calculated using the same ROI criteria across samples.

**TABLE 1:** Primary antibodies used for immunostaining.

Antibodies	Catalog No.	Source
Guinea pig anti‐RBPMS	A008712	Raygene
Mouse anti‐AP2	3B5	Developmental Studies Hybridoma Bank
Mouse anti‐Lin28a	MA1‐016	Invitrogen
Rabbit anti‐c‐Fos	2250S	Cell Signaling
Rabbit anti‐IBA1	10904‐1‐AP	Proteintech
Rabbit‐anti‐GFAP	RB‐087‐A0	Epredia

### 4.10 Quantification and Statistical Analysis

No animals or data points were excluded based on IOP response or RGC survival outcomes. Statistical analyses were conducted using GraphPad Prism (v10.6.1). Comparisons between two groups were assessed using two‐tailed Student's t‐tests, as appropriate. For analyses involving three or more groups, one‐way ANOVA was applied followed by Tukey's multiple comparisons tests. Significance was determined with P values less than 0.05, denoted as follows: ^*^0.01 ≤ *p* < 0.05, ^**^0.001 ≤ *p* < 0.01, ^***^0.0001 ≤ *p* < 0.001, ^****^ *p* < 0.0001, and “ns” for not significant. Detailed statistical information for all quantitative analyses is provided in Table S1.

## Author Contributions

S.R. and Q.W. conceptualized the study and designed the experiments. E.H. and Q.W. led the gene therapy experiments in vitro and in vivo. C.L. and W.L. contributed to the recordings. E.H., C.L., I.K., W.X., Z.W. and Q.W. performed glaucoma surgeries, intraocular pressure measurements, and immunohistochemistry. E.H., C.L., and I.K. assisted with tissue harvesting. E.H., Q.W., and S.R. wrote the manuscript with input from all authors.

## Ethics Statement

All animal procedures were reviewed and approved by the Institutional Animal Care and Use Committee of Binghamton University and were conducted in accordance with institutional guidelines and applicable regulations for the care and use of laboratory animals. The approved animal protocol number is 894‐23.

## Conflicts of Interest

The authors declare no conflicts of interest.

## Supporting information




**Supporting File**: advs78179‐sup‐0001‐SuppMat.docx.

## Data Availability

The data that support the findings of this study are available from the corresponding author upon reasonable request.
