# Supplementary material for: Modulation of Presynaptic Inhibitory Retinal Circuits for Retinal Ganglion Cell Neuroprotection in Glaucoma
Source: Adv Sci (Weinh). 2026 Oct 6:e78179. Online ahead of print. doi: 10.1002/advs.78179 (PMC13643093; doi:10.1002/advs.78179)
Supplement: Supplementary file 1 [file ADVS-9999-e78179-s001.docx]

**Modulation of Presynaptic Inhibitory Retinal Circuits for Retinal Ganglion Cell Neuroprotection in Glaucoma**

Eunji Hong^1^, Chen Lin^1^, Wenjie Long^1^, Ian Kim^1^, Mengwen Xiong^1^, Zuer Wu^1^, Siyuan Rao^1,2*^, Qianbin Wang^1,*^

^1^Department of Biomedical Engineering, Binghamton University, State University of New York, Binghamton, NY 13902, United States

^2^Integrative Neuroscience Program, Binghamton University, State University of New York, Binghamton, NY 13902, United States

Corresponding authors (*): Siyuan Rao (syrao@binghamton.edu) and Qianbin Wang ([qbwang@binghamton.edu](mailto:qbwang@binghamton.edu)).
**Table S1. Detailed statistical information for all quantitative analyses.**

| Figure number | Fig. 1E | |
| --- | --- | --- |
| Independent sample size | n = 4 | |
| Data presentation | mean ± SD | |
| Statistical test | One-way ANOVA | |
| Multiple comparison test | Tukey's multiple comparison test | |
| P value | Day 0 vs. Day 3 | P = 0.0853 |
|  | Day 0 vs. Day 7 | **P = 0.0094 |
|  | Day 0 vs. Day 10 | ****P < 0.0001 |
|  | Day 3 vs. Day 7 | P = 0.5974 |
|  | Day 3 vs. Day 10 | **P = 0.0042 |
|  | Day 7 vs. Day 10 | *P = 0.0386 |

| Figure number | Fig. 1K | |
| --- | --- | --- |
| Independent sample size | n = 5 | |
| Data presentation |  | |
| Statistical test | One-way repeated measures ANOVA | |
| Multiple comparison test | Tukey's multiple comparison test | |
| P value | Week 1 vs. Week 2 | P = 0.14 |
|  | Week 1 vs. Week 3 | *P = 0.0395 |
|  | Week 1 vs. Week 4 | *P = 0.0325 |
|  | Week 2 vs. Week 3 | P = 0.7111 |
|  | Week 2 vs. Week 4 | P = 0.4311 |
|  | Week 3 vs. Week 4 | P = 0.6689 |

| Figure number | Fig. 1L | |
| --- | --- | --- |
| Independent sample size | n = 5 | |
| Data presentation |  | |
| Statistical test | One-way repeated measures ANOVA | |
| Multiple comparison test | Tukey's multiple comparison test | |
| P value | Week 1 vs. Week 2 | P = 0.9782 |
|  | Week 1 vs. Week 3 | P = 0.9469 |
|  | Week 1 vs. Week 4 | P > 0.9999 |
|  | Week 2 vs. Week 3 | P = 0.942 |
|  | Week 2 vs. Week 4 | P = 0.9948 |
|  | Week 3 vs. Week 4 | P = 0.922 |

| Figure number | Fig. 2F | |
| --- | --- | --- |
| Independent sample size | n = 4 | |
| Data presentation | mean ± SD | |
| Statistical test | One-way ANOVA | |
| Multiple comparison test | Tukey's multiple comparison test | |
| P value | Saline-drug vs. Oil-drug | ***P = 0.0007 |
|  | Saline-drug vs. Oil+drug | P = 0.3742 |
|  | Oil-drug vs. Oil+drug | **P = 0.0047 |

| Figure number | Fig. 2H |
| --- | --- |
| Independent sample size | n = 5 |
| Data presentation | mean ± SD |
| Statistical test | Unpaired t-test |
| One- or two-tailed tests | Two-tailed test |
| P value | **P = 0.0042 |

| Figure number | Fig. 2J week 2 | Fig. 2J week 3 | Fig. 2J week 4 |
| --- | --- | --- | --- |
| Independent sample size | n = 5 | n = 5 | n = 5 |
| Data presentation | mean ± SD | mean ± SD | mean ± SD |
| Statistical test | Unpaired t-test | Unpaired t-test | Mann-Whitney test |
| One- or two-tailed tests | Two-tailed test | | |
| P value | P = 0.0935 | ***P = 0.0002 | **P = 0.0079 |

| Figure number | Fig. 2K Oil-drug | Fig. 2K Oil+drug |
| --- | --- | --- |
| Independent sample size | n = 5 | n = 5 |
| Data presentation | mean ± SD | mean ± SD |
| Statistical test | Kruskal-Wallis ANOVA | One-way ANOVA |
| Multiple comparison test | Dunn's multiple comparison test | Tukey's multiple comparison test |
| P value | Week 1 vs. Week 2 P = 0.6880 | Week 1 vs. Week 2 P = 0.4715 |
|  | Week 1 vs. Week 3 P > 0.9999 | Week 1 vs. Week 3 P = 0.0711 |
|  | Week 2 vs. Week 3 0.5373 | Week 2 vs. Week 3 *P = 0.0111 |

| Figure number | Fig. 3E | |
| --- | --- | --- |
| Independent sample size | n = 5 | |
| Data presentation | mean ± SD | |
| Statistical test | One-way ANOVA | |
| Multiple comparison test | Tukey's multiple comparison test | |
| P value | Vgat-EGFP vs. Vglut2-Lin28 | P = 0.2522 |
|  | Vgat-EGFP vs. Vgat-Lin28 | *P = 0.0396 |
|  | Vglut2-Lin28 vs. Vgat-Lin28 | **P = 0.0020 |

| Figure number | Fig. 3F | |
| --- | --- | --- |
| Independent sample size | n = 5 | |
| Data presentation | mean ± SD | |
| Statistical test | Kruskal-Wallis ANOVA | |
| Multiple comparison test | Dunn's multiple comparison test | |
| P value | Vgat-EGFP vs. Vglut2-Lin28 | P = 0.5373 |
|  | Vgat-EGFP vs. Vgat-Lin28 | P > 0.9999 |
|  | Vglut2-Lin28 vs. Vgat-Lin28 | P > 0.9999 |

| Figure number | Fig. 3I |
| --- | --- |
| Independent sample size | n = 5 |
| Data presentation | mean ± SD |
| Statistical test | Unpaired t-test |
| One- or two-tailed tests | Two-tailed test |
| P value | ***P = 0.0005 |

| Figure number | Fig. 3K |
| --- | --- |
| Independent sample size | n = 5 |
| Data presentation | mean ± SD |
| Statistical test | Unpaired t-test |
| One- or two-tailed tests | Two-tailed test |
| P value | **P = 0.0012 |

| Figure number | Fig. 3M |
| --- | --- |
| Independent sample size | n = 5 |
| Data presentation | mean ± SD |
| Statistical test | Unpaired t-test |
| One- or two-tailed tests | Two-tailed test |
| P value | **P = 0.0098 |

| Figure number | Fig. 4G | |
| --- | --- | --- |
| Independent sample size | n = 5 | |
| Data presentation | mean ± SD | |
| Statistical test | One-way ANOVA | |
| Multiple comparison test | Tukey's multiple comparison test | |
| P value | Before vs. Week 2 | *P = 0.0464 |
|  | Before vs. Week 4 | **P = 0.0033 |
|  | Before vs. Week 6 | P = 0.1228 |
|  | Before vs. Week 8 | *P = 0.0107 |
|  | Week 2 vs. Week 4 | *P = 0.0281 |
|  | Week 2 vs. Week 6 | P = 0.5165 |
|  | Week 2 vs. Week 8 | P = 0.1 |
|  | Week 4 vs. Week 6 | P = 0.9986 |
|  | Week 4 vs. Week 8 | P = 0.8481 |
|  | Week 6 vs. Week 8 | P = 0.3031 |

| Figure number | Fig. 4H | |
| --- | --- | --- |
| Independent sample size | n = 5 | |
| Data presentation | mean ± SD | |
| Statistical test | One-way ANOVA | |
| Multiple comparison test | Tukey's multiple comparison test | |
| P value | Before vs. Week 2 | P = 0.991 |
|  | Before vs. Week 4 | P = 0.9856 |
|  | Before vs. Week 6 | P = 0.9599 |
|  | Before vs. Week 8 | P = 0.9999 |
|  | Week 2 vs. Week 4 | P = 0.4838 |
|  | Week 2 vs. Week 6 | P = 0.3208 |
|  | Week 2 vs. Week 8 | P = 0.9105 |
|  | Week 4 vs. Week 6 | P = 0.9971 |
|  | Week 4 vs. Week 8 | P = 0.5353 |
|  | Week 6 vs. Week 8 | P = 0.1304 |

| Figure number | Fig. 4I | |
| --- | --- | --- |
| Independent sample size | n = 5 | |
| Data presentation | mean ± SD | |
| Statistical test | One-way ANOVA | |
| Multiple comparison test | Tukey's multiple comparison test | |
| P value | Before vs. Week 2 | P = 0.1877 |
|  | Before vs. Week 4 | P = 0.0848 |
|  | Before vs. Week 6 | P = 0.0842 |
|  | Before vs. Week 8 | *P = 0.0315 |
|  | Week 2 vs. Week 4 | P = 0.9924 |
|  | Week 2 vs. Week 6 | P = 0.9994 |
|  | Week 2 vs. Week 8 | P = 0.6 |
|  | Week 4 vs. Week 6 | P = 0.9847 |
|  | Week 4 vs. Week 8 | P = 0.1677 |
|  | Week 6 vs. Week 8 | P = 0.7839 |

| Figure number | Fig. 4J | |
| --- | --- | --- |
| Independent sample size | n = 5 | |
| Data presentation | mean ± SD | |
| Statistical test | One-way ANOVA | |
| Multiple comparison test | Tukey's multiple comparison test | |
| P value | Vgat-Lin28 vs. Vgat-EGFP | **P = 0.0022 |
|  | Vgat-Lin28 vs. Vglut2-Lin28 | **P = 0.0052 |
|  | Vgat-EGFP vs. Vglut2-Lin28 | P = 0.8764 |

**FigureS1:** Quantification of RGC survival before injection and at 1–4 weeks after viscobead injection.

**Figure S2:** Representative fluorescence microscopy images and quantification of retinal sections immunostained for GFAP and RBPMS in Vglut2-Lin28, Vgat-EGFP, and Vgat-Lin28 groups.

**Figure S3:** c-fos immunostaining (A) and *d*ERG analysis (B) assess ocular hypertension-associated INL neuronal hyperactivity across Vgat-EGFP, Vglut2-Lin28, and Vgat-Lin28 groups.

**Figure S4:** Quantification of daytime REM sleep before bead injection (A) and 8 weeks after bead injection (B).
